# Influencing factors in the implementation of postgraduate medical e-learning: a thematic analysis

**DOI:** 10.1186/s12909-019-1720-x

**Published:** 2019-08-05

**Authors:** Robert Adrianus de Leeuw, Daniel Nathan Logger, Michiel Westerman, Jochen Bretschneider, Marijn Plomp, Fedde Scheele

**Affiliations:** 1 VU Amsterdam, Athena Institute for Trans-Disciplinary Research, Boelelaan 1117, 1081 HZ Amsterdam, The Netherlands; 2 Franciscus Gasthuis & Vlietland Hospital, Department of internal medicine, Rotterdam, The Netherlands; 3 Amsterdam UMC, Vrije Universiteit Amsterdam, Amsterdam, The Netherlands; 4 VU Amsterdam, School of Business and Economics, Amsterdam, The Netherlands

**Keywords:** E-learning, Postgraduate medical education, Continuous education, Implementation, Innovation

## Abstract

**Background:**

Postgraduate medical e-learning (PGMeL) is being progressively used and evaluated. Its impact continues to grow, yet there are barriers to its implementation. Although more attention is now being paid to quality evaluation models, little has been written about the successful implementation of PGMeL. This study aims to determine factors and define themes influencing the successful implementation of PGMeL.

**Methods:**

We performed 10 semi-structured interviews with experienced e-learning creators, after which we carried out a thematic analysis to name and describe factors and themes.

**Results:**

Although this was not the objective of the study, the participants stressed the importance of a definition of success. Associated with this definition were: reaching your target audience, achieving learning aims, satisfying your audience and maintaining continuity. Three themes were identified containing eleven factors that influence successful implementation. The themes were named and defined after the group that had the most influence on the factors. We named them creator-, organization- and learner-dependent factors. The creator dependent factors are: the learning aim, pedagogical strategies, content expertise, evaluation and the creators motivational path. The organization dependent factors are management support, recourse and culture. Finally, the learner dependent factors are technology, motivators/barriers and value.

**Conclusions:**

This study shows that implementing PGMeL has creator-, organization- and learner-dependent factors which should be taken into account during the creating of the PGMeL. Although creator- and learner-dependent factors are mentioned in other studies, the present study also stresses the importance of organization-dependent factors. Innovation implementation theories such as Rogers’ diffusion of innovation or Kotter’s eight steps of change management show a great overlap with these factors. Future studies can both evaluate the use of these innovation models in creating PGMeL and assess the effect of the organizational factors in greater depth.

**Electronic supplementary material:**

The online version of this article (10.1186/s12909-019-1720-x) contains supplementary material, which is available to authorized users.

## Background

During the last decades electronic learning (e-learning) has been increasingly used in health professional education, and the number of e-learning modules has rapidly increased [1, 2]. To define e-learning we used Sangrà’s definition after a 2012 Delphi: “an approach to teaching and learning, representing all or part of the educational model applied, that is based on the use of electronic media and devices as tools for improving access to training, communication and interaction and that facilitates the adoption of new knowledge, skills and/or behavior/attitude” [3]. Many institutions are recognizing the power and benefits of internet-based learning, and they endorse learner-centered and personalized forms of learning [1, 4]. Previous studies show benefits in the educational experience and logistics (by overcoming traveling difficulties) and the increased interest can be seen throughout the continuum of medical education, specifically in postgraduate medical education [4, 5].

Despite the benefits, studies show that barriers to the implementation of postgraduate medical e-learning (PGMeL) remain [2, 4]. In 2001 a factor-analytic study provided a list of barriers [6] which was supported by a recent integrative review from 2018 [7]. These reveal barriers such as time constraints, poor technical skills and inadequate infrastructure by lack of technological basics such as intermittent internet or no email access. And although implementation is described as an important step in the process of using PGMeL efficiently [7–9], no studies have provided factors which influence the successful implementation of PGMeL. Although certain studies address evaluation and implementation as a whole, none of them discuss the details of the implementation [10].

Before we continue to discuss implementation, we will first provide our working definitions of implementation, creators and users. Implementation: the act of carrying an intention into effect, which in health research can be policies, programs or individual practices [11] or, specifically for PGMeL, the creation, management and delivery of learning content. Creator(s): the person/people who created the e-learning. This is usually a group of people with their own specialty (content, IT, education) [12]. User: the person who uses the e-learning to learn from it (the term “learner” is also used). In this study we focus on medical postgraduates. By postgraduates, we mean each person that has finished their graduate training and now work and learn at the same time. Earlier work shows that a different learning theory and pedagogical approaches can or should be used when targeting an adult audience [2]. Learning during work and therefore combining the responsibilities, working hours and stress of health care might be different from full-time learning. We choose therefore, to select a specific target audience as the users, as suggested good practice in current literature [13].

It can be argued that implementing a new innovation (in this case, an e-learning) implies change in an organization [14]. Two theories on change and innovation implementation have been used successfully to facilitate the use of new technology in health care: Rogers’ innovation diffusion theory and Kotter’s change management model [14–16]. While Rogers’ focusses on innovations as entities with attributes and a user decision process, Kotter describes an eight step change management process [17, 18]. Both models could be useful in the implementation of PGMeL, although there seems to be no literature describing their use in this regard.

Currently, the literature emphasizes the importance of implementation, and lists barriers to this. Some innovation implementation models are available, but only from other fields of expertise. The question this paper will try to answer is; which factors influence the successful implementation of PGMeL and how do such factors compare to these implementation models?

## Method

### Study design

**Fig. 1:**
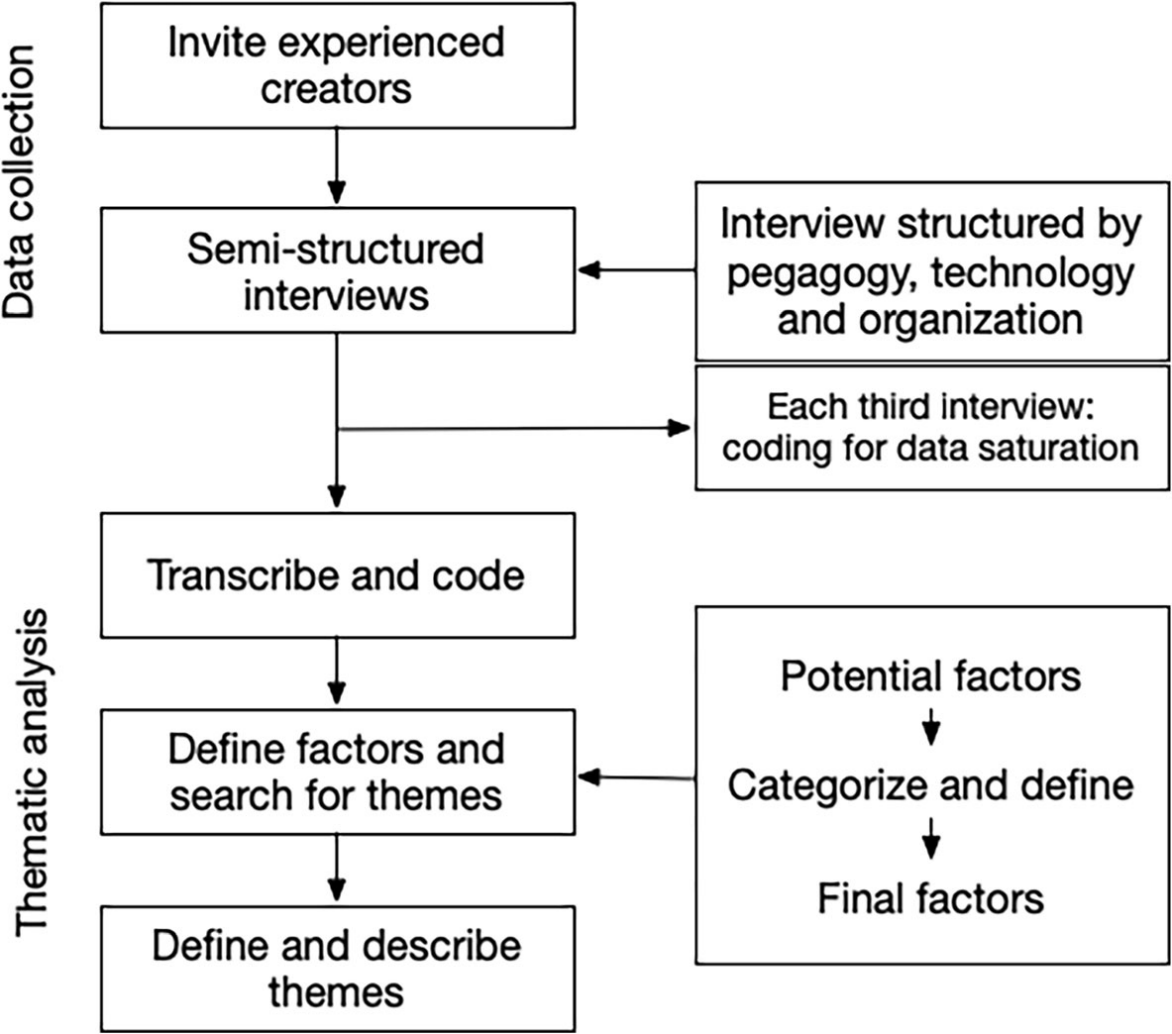
Flowchart of the thematic analysis performed in this study. The methodology for this study consists of; data collection by semi-structures interviews and thematic analysis by transcribing the data, categorize factors and define themes

We performed a series of semi-structured interviews with e-learning creators, after which we performed a thematic analysis in order to generate an answer to our question and categories of factors. The semi-structured format is a frequently used technique in qualitative healthcare studies. One of its main advantages is the interaction between interviewer and participant, which allows deeper insight into possible constructs [19]. See Fig. 1 for the flowchart of the applied methodology.

### Study participants

We invited experienced creators of PGMeL to participate in our study. We selected these creators from our professional network and through the national e-learning task group of the Dutch Association of Medical Education. These creators had to have experience of designing and implementing PGMeL for at least three years in collaborating with experts from different fields of medical specialties. No financial compensation was given to any participant. The ethical board of the Dutch Association of Medical Education gave ethical consent (2018.6.3), after which all participants gave their written informed consent. The interviews were in Dutch; therefore, citations are translated into English.

### Data collection method and procedure

All interviews were carried out in the Netherlands in the comfort of the participants’ own environment. The interviews lasted between 30 and 45 min and were facilitated by DL and audiotaped. The interview started with a short introduction regarding the reason for the interview, followed by a few demographic questions. The interview guide (Additional file 1) was developed by RL and DL and based on Lidgren et al. [20]. They grouped the implications of e-learning on pedagogy, technology and organization, which we followed in the interview guide. After each third interview the recordings were transcribed and coded. We continued interviewing experts in series of three until theoretical data saturation was reached.

### Data analysis

We verbatim transcribed all interviews and performed a thematic analysis according to Braun [21] in the original language. Transcribing of interviews was carried out by RL and DL. We used Atlas.ti version 8.0 for the initial coding. The reason for using thematic analysis is that is can usefully summarize key features and generate unanticipated insights, and has been proven useful for producing qualitative analysis suited to informed policy deployment [21]. To perform the data analysis in a structured method, we used the six steps proposed by Braun et al. [21], containing:

1. Familiarizing oneself with the data
2. Generating initial codes
3. Searching for themes
4. Reviewing themes
5. Defining and naming themes and
6. Producing the report.

### Coding and searching for themes (steps 2 and 3)

The second step was finding potential factors from the coded data. Coding by RL and DL led to 112 codes of which, after duplications and synonyms had been removed, 76 remained (see Table 1). To search for factors and themes, we wrote all codes on post-it notes and started arranging them on the wall. The first step was to categorize all factors from the codes, that were associated with the initial themes; pedagogy, technology and organization, followed by a mind-map of all factors. This led to the final factors. The mind-map, group discussion and digital evaluation of the mind-map followed, which gave insight into the underlying constructs behind the factors which we used to define the themes (see Fig. 3).

**Table 1:** Codes, factors and themes from the template analysis

Code	Factor	Domain
Target audience	Aim of the e-learning	Author dependent
Reaching target audience		
Learning aims		
Knowledge		
Skills		
Attitude/behavior		
Inefficiency		
Means to reach goal		
Learning methods	Pedagogical strategies	
Separate in sections		
Levels of knowledge		
Feedback		
Interactivity		
Communicate		
Type of content	Content knowledge	
Future evaluation	Evaluation	
Improvement		
Maintenance		
Reserve resources		
Technology centered	Motivational pathway	
Innovation driven		
Learner centered		
Author centered		
Needs driven		
Create commitment	Management support	Organization
Planning		Dependent
Responsibilities		
Insufficient work		
Deadlines		
Manage expectations		
Transparency		
Integrate into daily work		
Project leaders		
Motivate authors		
Provide support		
Reserve or provide time		
Promote project		
Involve stakeholders		
Include users		
ICT	Resources	
Team		
Knowledge		
Ongoing budget		
Technology budget		
Evaluation budget		
Management culture	Culture	
User culture		
Centralize		
Fragmentize		
Lack of structure		
Structure	Technology	Learner dependent
Design		
Infrastructure		
Availability		
Device variety		
Flexibility		
Layout		
Navigation		
Attractive		
Intuitive		
User friendly		
Motivate team	Motivators and barriers	
Motivate management		
Motivate users		
Not doing it		
Demotivating		
Ineffectiveness		
Promote		
External motivation		
Duraton		
Time efficiency		
Added value	Value	
Attractiveness		
Efficiency		
Time management		
Satisfaction		

Legend: By combing the original codes, the codes column in this table show key words associated with those codes. It demonstrates the content associated with the categories and themes

## Results

In the period May 2018 to August 2018 we invited a total of 14 expert creators. Four creators rejected the invitation due to lack of time. Ten interviews followed, after which data theoretical saturation was reached, and no further interviews were performed. On average the experts had eight years of experience (with an interval between three and twenty-five years). We interviewed six men and four women: six content experts and four with a management task concerning e-learning, of whom five were involved in research into different categories of e-learning. Participants were working in five different University Hospitals in the Netherlands. Step one of the thematic analysis, familiarizing oneself with the data, was performed during the transcribing. Citations from the interviewees are marked as “IT”, followed by a number corresponding with that person.

### Reviewing, defining and naming the themes (step 4 and 5)

**Fig. 2:**
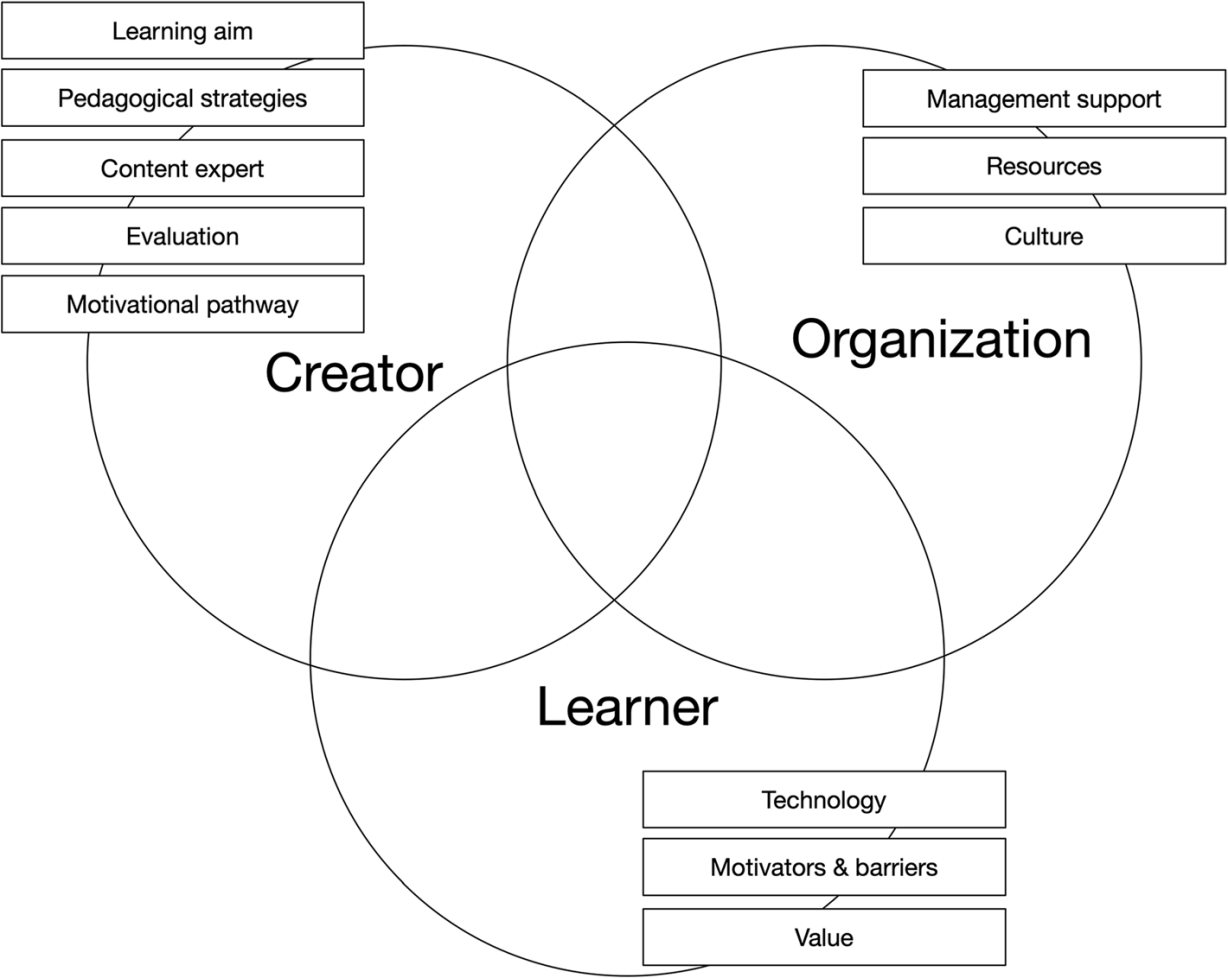
Themes and factors that influence the success of PGMeL implementation. Implementation success = (1) reach your target audience, (2) achieve learning goals, (3) create satisfaction and (4) provide continuity. The factors “Aim of the e-learning”, “Management support” and “Value” are concentrated within one theme. The other factors contain partially overlapping different themes, depending on the influence of creators, organization and learners

**Fig. 3:**
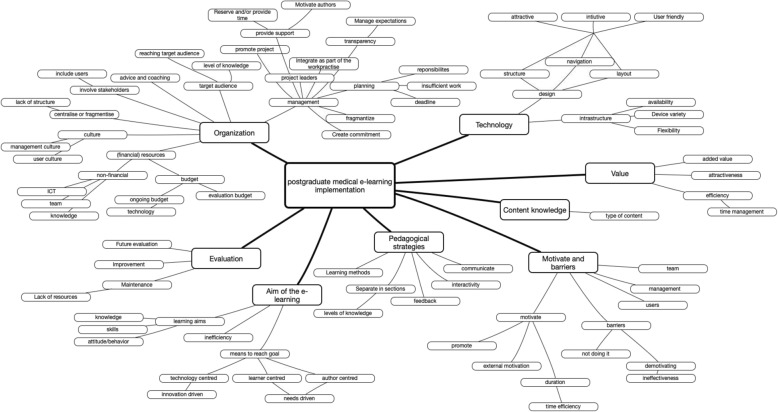
Mindmap of codes

From the data, we extracted eleven factors that were divided into three themes. Due to the blurred boundaries between the discussed subjects, some factors can fall within more than one theme. These factors were placed in the theme in which they, in theory, have the most influence. See Fig. 2 for all factors and themes.

The themes were named:

1. Creator-dependent factors
2. Organization-dependent factors
3. Learner-dependent factors

### Defining success

Although this was not the objective of this study, the participants stressed the importance of a definition of successful implementation of PGMeL. During the analysis, factors arose that concerned a possible aim for success. There were four aspects to this success:

1. It was suggested that for an implementation to be successful, the e-learning should be carried out/experienced by the right group of users (the target audience). An e-learning created for residents, but which is only carried out by interns is not properly implemented.
2. The e-learning should have defined learning goals, but users should be tested to see if they actually achieved those goals.
3. The users should have a satisfactory experience. The main importance of satisfaction is to provide a longer lasting learning experience and motivation for future e-learning experiences.
4. The e-learning needs continuity to maintain relevance and function as reference material for your users. To maintain relevance, the content should be updated if needed, and the technology remain accessible. An alternative is to provide a clear expiry date for the e-learning.

### Theme 1: creator-dependent factors

We defined this theme as *factors that the creators can largely influence,* in other words subjects that the creator team can discuss and make final decisions about. There were four factors within this theme:

1. Learning aim: what does the creator aim for the user to learn? Learning aims were grouped into knowledge, skills and attitude/behavior.
2. Pedagogical strategies: the learning instruments that are chosen to achieve the learning aim. There are several ways to achieve the learning aim and there is an added value to choosing a pedagogical strategy: *“using and consulting educational experts are important to reach your goals” IT6.* Certain strategies seem less relevant, although several items of these strategies were mentioned, including interactivity, feedback and segmentation: *“e-learning needs a sound and responsible pedagogical method, although there might be many of these” IT3.*
3. Content expert: having at least one person with in-depth knowledge of the subject of the e-learning. Having a content expert is reported as very relevant to implementation. Having expert content knowledge available at all stages of the design and, for example, during meetings helps in discussion of relevance and importance. Moreover, although content experts can be the driving force behind the e-learning, creating PGMeL is a team effort and delegating tasks can improve efficiency: *“it can help for a content expert to draft the major lines, so others can continue working on it to create an e-learning” IT5*
4. Evaluation: a part of the success of implementation lies in the continuity of the e-learning. Evaluation is needed to continue improving the e-learning, but also to collect alarm signals for bugs, outdated sources and lack of resources for the future.
5. Creators motivational pathways: the interviewees described the different ways in which they thought important decisions around creating PGMeL were initiated and motivated. We called this the “creators motivational path”. They described three different perspectives: e-learning can be technology-, learner- and creator-centered. Centeredness means why the e-learning was created in the first place and what motivated the creators to make the e-learning. Technology-centered e-learning aims to use and evaluate a new technology which a creator feels should be used for teaching: *“IT departments innovate faster (than education departments) and technology enhanced learning can anticipate on educational needs” IT7.* Learner-centered e-learning aims to fulfil the needs of a learner, by means of technology. A potential student, or groups of students, have the need to learn something and a content expert searches for the right tool to achieve that goal. Creator-centered e-learning aims to fulfil the need of a creator to spread something new or educate a certain group. This creator will then make the e-learning, after which she/he will start sharing it and search for an audience: *“Authors can feel this urge (to create PGMeL), which can lead to new creations” IT2.*

All three forms have their own pitfalls and possibilities (see Table 2) to be considered when implementing PGMeL.

**Table 2:** Pitfalls and possibilities of the motivational paths of the creators

Group	Pitfalls	Chances
Technology-centered	No clear learning aims	Evaluation of new technology
	Risk of evaluation without control group	Development of next technologies
	No initial user or creator support	Investment possibilities
Learner-centered	Possible lack of innovation	Needs come from the learner
	Need to motivate creators	Need for improvement of available tools
		Available learner commitment
Creator-centered	Risk of no learner need	Motivated creators
	Possible lack of innovation	New knowledge shared

Legend Table 2: a motivational path is the reason which motivated the e-learning creators to make the e-learning. They can be motived by a new technology that they want to test (technology-centered), because there is a need from a learner’s perspective (learner-centered) or from the need of the creators themselves to share of educate a lesson (creator-centered).

### Theme 2: organization-dependent factors

We defined this theme as *factors largely influenced by the organization where the e-learning is being used.* This theme contains three factors:

1. Management support: the organization contains many management aspects such as planning, having project leaders, receiving commitment from higher management, transparency and managing expectations. However, organization also means involving stakeholders, and knowing and reaching your target audience. Support from all these parts of the organization is preferred.
2. Resources: several topics were discussed around resources. There are non-financial resources, such as an ICT department, team support and knowledge support, and there are financial resources, which emphasize an ongoing budget for maintenance, a technology budget and an evaluation budget.
3. Culture: implementation also requires a certain culture, that is, a management culture to provide the needed support, but also a user culture that is willing to learn and evaluate with you.

### Theme 3: learner-dependent factors

We defined the theme as *factors that are mainly influenced by the learner,* and by learner we mean the end-user of the e-learning. Three factors fall within this theme:

1. Technology: this was mentioned as an important aspect of the design and the infrastructure. Design elements were mentioned in the navigation, structure of the e-learning and layout. It should be attractive, intuitive and user-friendly. A proper design helps with the implementation, but the infrastructure of the e-learning is just as important: *“it has to be as user-friendly as possible; nobody likes a socket that does not fit a plug” IT8.* By infrastructure we mean the way the e-learning is made available, device compatibility and user flexibility (availability anytime, anywhere on any device, adjustable to the user’s personal needs). The technology is usually chosen and used by the creator and depends on the needed affordances. Technology also needs support from the organization, but it is the learner who experiences and uses it to learn. Therefore, we believe that the learner has the most influence on the added value of technology to the learning process.
2. Motivators and barriers: the success of your implementation will depend on how you can motivate: *“to motivate (your students), you need to keep stimulating them so they (the students) will finish it” IT10.* Even when motivation is high, however, barriers which might lead users to not start in the first place must be prevented. The organization and creator both have an influence on these factors, but we believe the learner has the upper hand.
3. Value: the learner must experience added value from the e-learning over other forms of education or already existing learning material. The added value should be clear for the user and can be found in attractiveness and efficiency: *“it really needs additional value, compared to (for example), books” IT3.*

## Discussion

This study provides eleven factors in three themes; learner, author and organization dependent factors. The learner depend factors technology, motivators and barriers and added value have previously been discussed as important aspects [2, 22, 23] and much has been written about this. This paper emphasis these aspects, yet, adds little new insight.

Of the author dependent factors, the importance of considering and following a pedagogical strategy has been preached for a long time [7], although it is still frequently ignored [24]. Defining the aim of the e-learning and the evaluating the results, are also suggested in other studies [22]. However, considering the creators motivational path does provide new insight. Studies have stressed the importance of user-centered e-learning [25], yet this study shows that there are other motivators that initiate the creators need for making PGMeL, all of which present pitfalls and opportunities.

The series of organization-dependent factors is much less frequently discussed in the literature [23, 26, 27]. Commitment is needed from an organizational standpoint (that is, whether management grants time, resources and merit). Management support will help to give substance to deadlines, expectations and agreements. If the organization advocates the product, this will also help with changing the culture. Financial resources are also an undervalued topic [27], and this is even more the case for the non-financial resources needed. This study aims to place more emphasis on these topics for future research.

### Success in implementation

Defining success as reaching your target audience, achieving learning aims, satisfying your audience and maintaining continuity is a good starting point. However, success should be defined for each individual project. The most important message is that success should be defined before implementation. These four factors are a starting point only, on which future research can build.

### PGMeL implementation as innovation implementation

Considering PGMeL in an organization as an innovation, it makes sense to use innovation implementation models. Rogers’ model of diffusion of innovation and Kotter’s eight step model have a significant overlap with the factors addressed in this study. In Table 3, three aspects of Rogers’ model of diffusion of innovation are summarized: the decision steps, the attributes of innovation and the adopter categories. In the second column, the interpretation of these steps from the perspective of this study are added. The models start with an innovation, which is the e-learning. The creators (the source) will then communicate the existence of the e-learning through the organization (channel). The users will go through a decision process to determine if they accept the e-learning. This process has five stages and after stage three the user will decide whether to start the e-learning or reject it. The chances of acceptance depend on a series of attributes. Rogers’ model emphasizes the importance of the organization in terms of communication, time and social system. The attributes of relative advantage (value), compatibility (technology), complexity (adjustment to target audience), trialability (motivators and barriers) and observability (value and culture) are all mentioned in this study as well.

**Table 3:** Interpretation of Rogers’ diffusion of innovation for PGMeL

Step	Rogers stage	PGMeL equivalent
Decision process		
1	Knowledge	inform users of existence
2	Persuasion	convince users of importance
3	Decision	start or reject the e-learning
4	Implementation	reach the target audience
5	Confirmation	evaluate the learning aims
Attributes of the innovation		
1	Relative advantage	added value
2	Compatibility	design should fit the organization
3	Complexity	adjust to target audience
4	Trialability	personalize learning path
5	Observability	results should be visible
Adopter categories		
1	Innovators	creator team
2	Early adopters	pilot group
3	Early majority	first users
4	Late majority	last users
5	Lagers	unwilling

Legend Table 3: Rogers distinguishes three aspects of innovation implementation. The decision process of the user, the attributes of the innovation and the categories of users called adopters. The decision process contains five steps that the user will go through. Those steps can be translated into PGMeL, shown in the last column. The attributes should be linked to the created e-learning, making it more likely that the user will decide to accept the e-learning. Finally, defining the users/adopter factors might help identify barriers for each group.

As regards Kotter’s eight steps model, Table 4 shows an interpretation of these steps within PGMeL implementation. Kotter distilled his principles of change into these eight mandatory steps. Quinn et al. used this model before to re-engage student into blended learning and found gaps, in particular to the support for students [28]. The eight steps also only show an overlap with a part of the factors from this study. Kotter is developed and aimed at corporate change, and emphasis importance to organizational efforts like establishing urgency, creating a coalition, communicate, empower others and create cultural changes. This study adds specific factors for education and placed more responsibility with the learner as well. Comparing the themes and factors addressed in this study with those in the two innovation models supports the importance of most factors and shows that neither model is as specific to PGMeL as the outcome of this study.

**Table 4:** Interpreting Kotters’ eight steps for PGMeL

Step	Original description	PGMeL interpretation
1	Establish a sense of urgency	Why you need this e-learning
2	Create a guiding coalition	Create your optimal team
3	Develop a change vision	What the e-learning will establish
4	Communicate that vision	Communicate these aims
5	Empower others to act	Empower users
6	Garner short-term wins	Define short-term wins
7	Never let up	Determine a strategy when resistance is shown
8	Incorporate changes into culture	Incorporate continuous, digital learning into culture

Legend Table 4: Kotters eight steps can be interpreted for PGMeL, by rewriting them into questions or advice for the creators, shown in column three. Kotters model is focused more on the organizational aspects of implementation, therefore, takes little to no aspects of medical education into account.

### Strengths and limitations

One of the strengths of this study is the overlap with existing literature. Many subjects have previously been described and every time a group of experts overlaps previous knowledge, the path we are following is reinforced. This study broadens our scope and retrieves far less discussed topics, such as the organizational aspects of PGMeL. A diverse group has been interviewed; whose variety of expertise enforces the backbone of this study. However, there are also limitations, the biggest of which is the bias in selection. It is impossible to say whether the balance between management, content and technological experience within the group of interviewees is correct or over-emphasizes a single subject. Moreover, in studies like this, there is always the issue of cross-cultural generalizability, as all participants are from a Western culture and function and create PGMeL within these limits. Organizational aspects that are relevant in Western culture might be completely different in Eastern cultures. It may even be argued that every organization has its own sub-culture and for that reason also has different needs. To minimize that limitation, we included participants from different regions in the Netherlands, but the fact remain that they are all from the Netherlands.

### Future research

Some of the factors addressed in the present study are already receiving due attention but much remains to be learnt about the organizational needs for creating PGMeL. Involving more stakeholders from management level might provide more insight into the limitations of an organization. Using models such as the innovation of diffusion provide insight as to where a specific e-learning might experience implementation barriers and which factors could be improved. Evaluating the use of these factors and models in practice would be of great value to keep adjusting and improving them. This study also provides a starting point for defining success.

## Conclusion

This study shows that implementing PGMeL has creator-, organization- and learner-dependent factors. These factors influence the success of the implementation. To achieve successful implementation, you must reach your target audience, achieve learning aims and provide continuity. Factors that will help achieve that success are partly outside the creators’ influence, and partly within it. The organization-dependent factors, management support, resources and culture, might be those most in need of attention to achieve a well-earned implementation success, since relatively little is known and written about them. This study also shows that, in theory, using innovation and change management models could be very helpful, and certainly merits further research.

### Additional files

Additional file 1 Interview guide (DOCX 13 kb)

## Data Availability

The datasets used and/or analyzed during the current study are available from the corresponding author on reasonable request.

## Abbreviation

PGMeL: PostGraduate Medical E-learning
