# Supplementary material for: Influencing factors in the implementation of postgraduate medical e-learning: a thematic analysis
Source: BMC Med Educ. 2019 Aug 5;19:300. doi: 10.1186/s12909-019-1720-x (PMC6683402; doi:10.1186/s12909-019-1720-x)
Supplement: Supplementary file 1 — Interview guide (DOCX 13 kb) [file 12909_2019_1720_MOESM1_ESM.docx]

# Appendix 1 – interview guide

**Introduction**

We met today to discuss the implementation of postgraduate medical e-learning together. By e-learning we mean every form of electronic / digital learning aimed at people who have to learn further alongside the working life. We strive today to explore what the goal is of the implementation of "postgraduate medical e-learning" (PGMeL) and what tools can be offered to achieve this goal. The interview will last about 30 minutes to an hour.

**A. Interview background**

Can you tell us something about your current position and your experience with e-learning and its implementation?

**B. The purpose of implementation**

In your view, what is the purpose of e-learning in a general sense?

How does this goal relate to the implementation of e-learning?

What do you understand by successful implementation of e-learning

**C. Domain technology**

What is the role of technology in implementation?

**D. Domain organization**

What is the role of an organization in the implementation?

What do you think of the responsibilities of the organization and management in the implementation and what have you experienced or missed in the past?

**E. Domain pedagogy**

What is the role of pedagogy in the implementation?

**F. General remarks**

Many thanks for answering these questions. Are there any general things that you think are of added value to include in the implementation of PGMeL?
